# The mediating role of coping styles between nurses’ workplace bullying and professional quality of life

**DOI:** 10.1186/s12912-023-01624-y

**Published:** 2023-12-05

**Authors:** Rui Jiao, Jinping Li, Nan Cheng, Xiangying Liu, Yizhou Tan

**Affiliations:** 1 Department of Nursing, Wuhan University of Science and Technology, Wuhan, Hubei China; 2 Hubei Province Key Laboratory of Occupational Hazard Identification and Control, School of Medicine, Institute of Nursing Research, Wuhan University of Science and Technology, Wuhan, Hubei China

**Keywords:** Nurses, Professional quality of life, Workplace bullying, Coping styles, Mediating effect

## Abstract

**Aims:**

This study aimed to explore the relationship between workplace bullying among nurses and their professional quality of life, as well as the mediating role of coping styles between the two factors.

**Background:**

In China, the overall status of nurses' professional quality of life is not optimistic, and the problems of low compassion satisfaction and high compassion fatigue persist. Workplace bullying, which is a serious global issue, can negatively impact the mental health and professional quality of nurses. However, it has still not attracted enough attention from managers.

**Methods:**

The study used a cross-sectional research design and surveyed 297 clinical nurses from two tertiary grade A hospitals in Wuhan, China. Data were collected through an online questionnaire survey from March to May 2022. The data were analyzed using descriptive statistical methods, including Pearson correlation analysis and structural equation modeling.

**Results:**

The score for nurses' workplace bullying was 38.72 ± 12.30. The scores for the three dimensions of professional quality of life were 27.56 ± 4.79 for compassion satisfaction, 30.51 ± 4.33 for burnout, and 28.47 ± 4.65 for secondary trauma stress. The scores for positive coping style and negative coping style were 34.59 ± 5.72 and 20.34 ± 5.08 points, respectively. Workplace bullying had a direct negative effect on compassion satisfaction, as well as positive direct effects on burnout and secondary traumatic stress. Coping styles played a mediating effect between workplace bullying and the pairwise relationships of compassion satisfaction, burnout, and secondary trauma stress.

**Conclusion:**

Workplace bullying hurts nurses' professional quality of life while coping styles plays an mediating role between workplace bullying and professional quality of life. Nursing managers can improve nurses' professional quality of life by reducing workplace bullying and enhancing positive coping style.

**Implications for nursing management:**

Nursing managers can employ management wisdom and techniques to mitigate the presence and detrimental effects of workplace bullying. This, in turn, promotes a positive work environment and enhances the professional quality of life for nurses.

## Introduction

With the continuous evolution of the nursing profession and the occurrence of public health crises worldwide, nurses' work content and status are constantly being challenged. As a profession that assists, the professional quality of life (ProQOL) of nurses refers to the quality one feels in the process of providing nursing services to patients [1, 2]. ProQOL is a multidimensional concept that includes positive compassion satisfaction and negative compassion fatigue, which is made up of burnout and secondary traumatic stress [3, 4]. The nurses' ProQOL is linked to the characteristics of their work environment, their traits, and their sensitivity and response to work pressure [5]. Therefore, the nurses' ProQOL is a dynamic and comprehensive concept [2].

According to worldwide research, the ProQOL of nurses is not optimistic, and the situation of low levels of compassion satisfaction and high levels of compassion fatigue is common [6]. Previous studies have shown that burnout can lead to physical and mental problems among nurses, such as insomnia, headache, difficulty focusing, long-term fatigue, and irritability [7]. It can therefore lead to a decline in the quality of nursing service and patient satisfaction, an increase in the turnover rate of nurses, and negative patient outcomes such as medical error, malpractice, and higher morbidity and mortality rates [7, 8].

### Background

Workplace bullying was first put forward by Meissner in 1986. It refers to harassment, offense, social exclusion, and other negative behaviors related to work tasks [9]. The negative behaviors are recurrent and long-term. It is manifested by repeated occurrences every week or more frequently, lasting for six months or more [10, 11]. Bullying in the care industry has been and will continue to be, a phenomenon for many years, according to a worldwide literature review [12]. Nurses who are bullied involve some common characteristics, such as being intimidated, humiliated, ignored, and isolated from colleagues, with work information being withheld, and professional status being compromised [13]. Compared with other medical service providers (such as doctors and therapists), nurses have a higher incidence of workplace bullying [12]. Studies in different countries showed that 39.1%-65.8% of nurses had reported workplace bullying [14, 15], and it was 52.1% showed by a study on Guangzhou nurses in China [16]. However, it is worth noting that a comprehensive report pointed out that Asian countries influenced by Confucianism are more accepting of work-related bullying than countries in Britain, America, Latin America, and Sub-Saharan Africa, which may affect the emergence or maintenance of this behavior [17]. Due to a lack of awareness about workplace bullying, nurses may not be able to clearly identify and cope with it correctly [18].

According to the victims’ report, long-term bullying and persecution had a range of physical, emotional, and psychological knock-on effects, such as anxiety, sleep disruption, trauma, helplessness, powerlessness, silence, anger, depression, and post-traumatic stress disorder. Long-term exposure may also lead to physical effects such as decreased immunity, stress, headaches, high blood pressure, and digestive problems [13]. For the nursing profession, persistent bullying will seriously damage professional values and the quality of life of practitioners. This can lead to a decline in job satisfaction and an increase in burnout, causing some individuals to leave the industry, and shaking the stability of nursing teams [19, 20].

The negative effects and pressures of workplace bullying motivate nurses to respond differently. Coping styles refers to an individual’s flexible, purposeful, and conscious regulation in the face of changes in the natural environment. It is an internal factor for individuals that can be categorized as either negative or positive [21]. In the workplace, those who take positive countermeasures can produce positive emotions and behaviors, leading to improved job satisfaction and a greater willingness to remain in their positions [22]. Meanwhile, those who take negative countermeasures will produce negative thoughts and evasive behaviors, and doubt their work ability, thus reducing their ProQOL [23, 24].

Adopting a negative coping style at work can easily lead to job burnout. On the contrary, job burnout is reduced when an individual has a clear attitude and can adopt a positive one frequently [25]. That is to say, coping styles is an mediating variable between job stress and burnout. Previous studies have proven that workplace bullying has a negative impact on nurses’ compassion satisfaction and has a positive impact on compassion fatigue [16], while coping styles have a significant predictive effect on nurses' ProQOL [26]. Up to now, no reports have been found regarding the mediating effect of coping styles between workplace bullying and nurses' ProQOL. Therefore, this study aims to verify the mediating effects of coping styles between workplace bullying and ProQOL among nurses by constructing structural equation models.

Study assumptions are as follows:

- Hypothesis 1: Workplace bullying is negatively and directly related to compassion satisfaction;
- Hypothesis 2: Workplace bullying is positively and directly related to burnout and secondary trauma stress;
- Hypothesis 3: Positive coping style mediate the relationship between workplace bullying and ProQOL.
- Hypothesis 4: Negative coping style mediate the relationship between workplace bullying and ProQOL.

**Methods**

### Study design and participants

The convenience sampling method was adopted in this study, and clinical nurses from two tertiary grade A hospitals in Wuhan, Hubei Province, were selected as the research subjects from March to May 2022. The inclusion criteria for the research subjects were as follows: being above 18 years old, holding a valid nurse qualification certificate and registered practice certificate, having worked in the hospital for more than 6 months, being directly involved in the clinical nursing of patients, and voluntary participation in the study. Exclusion criteria included nurses on leave during the investigation period and interns or refresher nurses. Based on the current survey's sample size calculation formula, *n* = (Ζ_α/2_σ/δ)^2^ [27], the sample size was determined with a standard deviation σ = 15, an allowable error δ = 2, and an α = 0.05 bilateral. Considering a shedding rate of 20%, the final sample size was determined to be 260 cases. The guiding principle for the optimal sample size of the structural equation model (SEM) is a minimum of 200 cases [28]. This study ultimately included 297 nurses.

### Instruments

#### General information questionnaire

The questionnaire was compiled by the researchers and included questions about gender, age, educational background, working years, department, professional title, employment type, daily working hours, monthly night shifts, income level, and marital status.

### The Negative Acts Questionnaire Revised (NAQR)

The scale was compiled by Einarsen [29] and revised by Hongjing Xun [30]. There were 22 items in the three dimensions, including 9 items related to personal negative behavior, 9 to work negative behavior, and 4 to organizational injustice. The scores ranged from "never" to "every day" based on the Likert-5 point scale. The total score on the scale ranged from 22 to 110, with a higher score indicating more severe workplace bullying. The Cronbach's α value of the Chinese version of the scale was 0.915. And the Cronbach's α value of the scale in this study was 0.937.

### Professional Quality of Life Scale (Pro-QOL)

The scale was compiled by Stamm [2] and revised by Xing Zheng [31]. It includes three dimensions: compassion satisfaction, burnout, and secondary trauma stress, each dimension containing 10 items. The scores range from 1–5 points for "Never had" to "Always had this" based on the Likert-5 level scoring system. Items 1, 4, 15, 17, and 29 are scored reversely, while the rest are scored positively. The three dimensions are scored separately, and scores of each dimension ≤ 22, 23–41, and ≥ 42 are considered to be at the low, medium, and high levels, respectively. The Cronbach's α value of the Chinese version of the scale was 0.710. And the Cronbach’s α value for this scale in this study was 0.782.

### The Simplified Coping Style Questionnaire (SCSQ)

The scale was compiled by Ya’ning Xie [32] and contains 20 items. The positive coping dimension consists of items 1–12, and the negative coping dimension consists of items 13–20. The scoring system is based on the Likert-4 level, with scores ranging from "Never used" to "Frequently used" and values of 0 to 3, respectively. A higher positive coping score indicates a greater likelihood of adopting a positive coping style, while a lower score suggests a tendency towards negative coping. The Cronbach's α value of the Chinese version of the scale was 0.900. And the Cronbach’s α value for this scale in this study was 0.915.

### Data collection

The investigation was carried out during the period of COVID-19 control in China. Due to this, we opted for an online method instead of a face-to-face paper questionnaire. Before data collection, we contacted the directors of the nursing departments of two hospitals to explain the purpose and details of this study and obtained their consent and support. The link to the online questionnaire was then distributed to the WeChat group of the above two hospitals, ensuring that the inclusion and exclusion criteria were clarified. Nurses who met the inclusion criteria were invited to fill out the questionnaire. The preface of the questionnaire told the participants the purpose of this study and the approximate time required to complete the questionnaire. Each IP address and device were limited to one submission. After the investigation, the data were reviewed by two nursing graduate students who had studied and been trained, and questionnaires with regular answers or completion times of less than 3 min were excluded. The final data underwent a thorough check by a master tutor. A total of 306 questionnaires were filled out and submitted for this study, and a total of 297 questionnaires have been deemed valid, resulting in an effective recovery rate of 97.06%.

### Data analysis

For data analysis and structural equation modeling, we used IBM SPSS Statistics 26.0 and AMOS 26.0. The demographic and medical features of the participants, as well as their levels of workplace bullying, professional quality of life, and coping styles, were described by mean and standard deviation, frequency, and percentage. The relationship between workplace bullying, professional quality of life, and coping styles was investigated using Pearson correlation analysis. We built the SEM using AMOS 26.0 and used full information maximum likelihood to estimate the relationships and parameters between variables. We investigated the mediating effect of coping styles on workplace bullying and professional quality of life with 5000 bootstrap resamples to test and validate the mediation effect, adopting 95% confidence intervals (CI) to test the direct and indirect effects. The inspection level was set at α = 0.05.

### Ethical considerations

This study has been approved by the Ethics Review Committee of the Medical College of Wuhan University of Science and Technology (approval number: 2022103) and adhered to the Declaration of Helsinki. Each subject provided informed consent and participated voluntarily, with the right to withdraw from the investigation at any time without impacting their work or personal life. Participant information was protected anonymously, and the contents of the questionnaire were used solely for the purpose of this study and would not be disclosed or leaked to any unauthorized parties. The research team ensured adherence to national and international ethical principles and codes of conduct throughout the study.

## Results

### Demographic characteristics

A total of 297 nurses were included in the study, comprising 22 males (7.4%) and 275 females (92.6%). Among the investigated nurses, 204 (68.7%) had less than five years of work experience, 263 (88.6%) were under 35 years of age, and 230 (77.4%) had a bachelor's degree or above. 164 nurses (55.2%) worked more than 8 h a day, with 44.4% earning less than 5,000 yuan and 55.6% earning more than 5,000 yuan. 203 (68.3%) were unmarried, and 94 (31.7%) were married. The general data is shown in Table 1.

**Table 1:** Demographic characteristics (*N* = 297)

Variables		*N*	(%)
Gender	Male	22	7.4
	Female	275	92.6
Age (year)	≤ 25	157	52.9
	26–35	106	35.7
	≥ 36	34	11.4
Working years	≤ 5	204	68.7
	6–10	49	16.5
	≥ 11	44	14.8
Educational level	College	67	22.6
	Undergraduate	175	58.9
	Master’s degree and above	55	18.5
Professional title	Junior nurse	139	46.8
	Senior nurse	103	34.7
	Nurse-in-charge and above	55	18.5
Work department	Internal medicine	87	29.3
	Surgical department	66	22.2
	Critical care department	26	8.8
	Gynecology, obstetrics and Pediatrics department	23	7.7
	Others	95	32.0
Daily working hours	≤ 8	133	44.8
	9–12	144	48.5
	> 12	20	6.7
Monthly night shift	0–4	143	48.1
	5–9	114	38.4
	> 10	40	13.5
Income(RMB)	< 5000	132	44.4
	5000–10000	138	46.5
	> 10000	27	9.1
Marital status	Unmarried	203	68.3
	Married	94	31.7

### Testing of common method deviations

Using the Harman univariate test, all items in the workplace bullying, ProQOL, and coping style scales were analyzed with non-rotating exploratory factor analysis. The results showed that 16 common factors with feature values > 1 were extracted. The explanation rate of the first common factor on the total variables was 22.29%, which was lower than the judgment standard of 40% [33]. This indicated that there was no significant common method deviation in the data of this study.

### Means, standard deviations, and correlations between major variables

The workplace bullying score of the 297 nurses was (38.72 ± 12.30). The scores for all dimensions of ProQOL were as follows: compassionate satisfaction (27.56 ± 4.79), burnout (30.51 ± 4.33), and secondary trauma stress (28.42 ± 4.65). Positive and negative coping style were (34.59 ± 5.72) and (20.34 ± 5.08), respectively. Pearson correlation analysis showed that workplace bullying had a negative correlation with compassion satisfaction and a positive correlation with burnout and secondary trauma stress. Positive coping style was positively correlated with compassion satisfaction and negatively correlated with burnout and secondary trauma stress. Negative coping style was negatively correlated with compassion satisfaction and positively correlated with burnout and secondary trauma stress. Details of information are shown in Table 2.

**Table 2:** Means, standard deviations and Correlations between major variables

Variables	Mean (SD)	1	2	3	4	5	6	7	8	9
1.WPB	1.74(0.56)	1	-	-	-	-	-	-	-	-
2. PRN	1.67(0.56)	0.949^**^	1	-	-	-	-	-	-	-
3. WRN	1.72(0.61)	0.954^**^	0.877^**^	1	-	-	-	-	-	-
4. OI	2.08(0.76)	0.773^**^	0.619^**^	0.621^**^	1	-	-	-	-	-
5. CS	2.76(0.48)	-0.425^**^	-0.417^**^	-0.362^**^	-0.386^**^	1	-	-	-	-
6. BO	3.06(0.43)	0.487^**^	0.454^**^	0.440^**^	0.435^**^	-0.388^**^	1	-	-	-
7. STS	2.86(0.46)	0.447^**^	0.404^**^	0.430^**^	0.374^**^	-0.263^**^	0.709^**^	1	-	-
8. PCS	2.85(0.47)	-0.488^**^	-0.499^**^	-0.441^**^	-0.363^**^	0.469^**^	-0.523^**^	-0.422^**^	1	-
9. NCS	2.55(0.64)	0.558^**^	0.518^**^	0.518^**^	0.478^**^	-0.415^**^	0.505^**^	0.492^**^	-0.408^**^	1

① *WPB* Workplace bullying

② *PRN* Person-related negative

③ *WRN* Work-related negative

④ *OI* Organizational injustice

⑤ *CS* Compassion satisfaction

⑥ *BO* Burnout

⑦ *STS* Secondary trauma stress

⑧ *PCS* Positive coping style

⑨ *NCS* Negative coping style

### Results of a hypothetical structural equation model

#### Construction of an mediating effect model

**Fig. 1:**
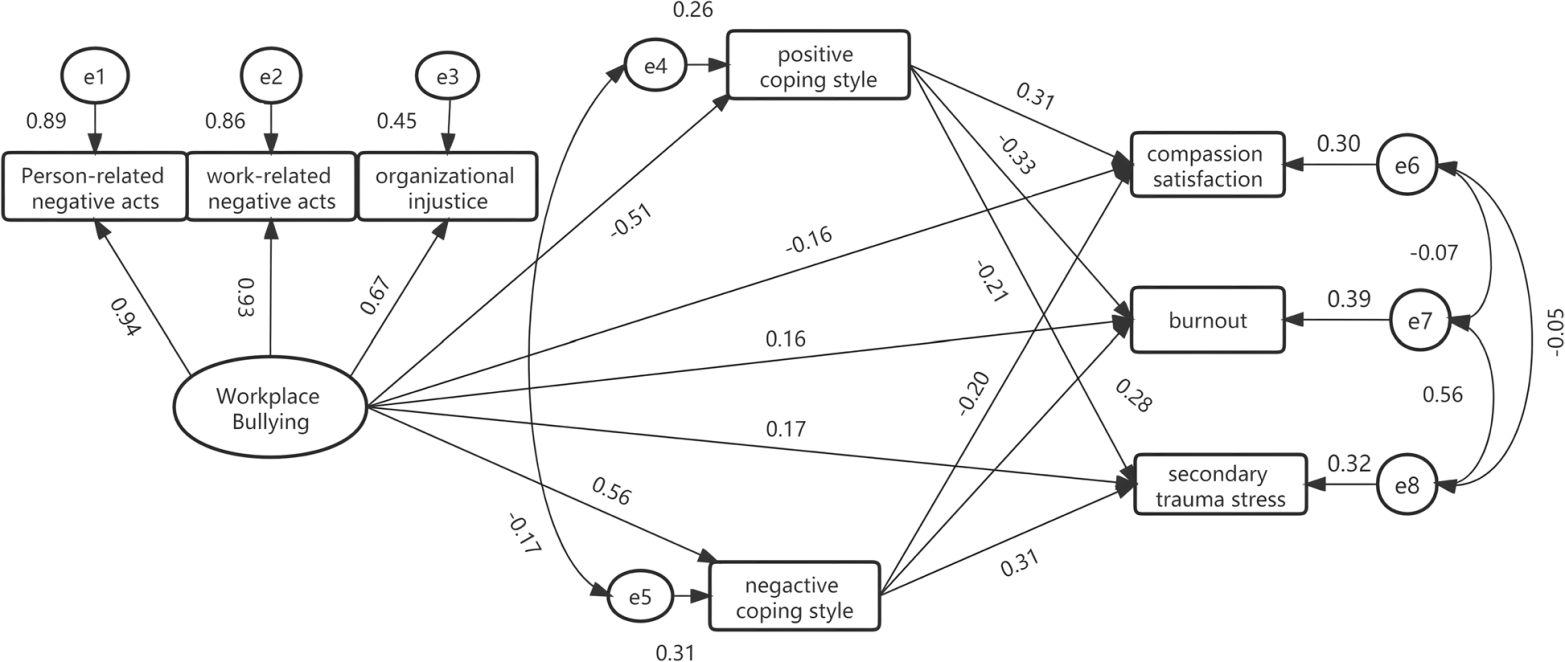
The mediation model of coping styles between nurses' workplace bullying and ProQOL (standardized)

Based on the correlation analysis results, a SEM was constructed. Workplace bullying was the independent variable, coping styles was the mediating variable, and all dimensions of ProQOL were the dependent variable, as shown in Fig. 1. The structural equation was corrected and fitted using the maximum likelihood method. The result of the model fitting was as follows: chi-square/degree of freedom (χ^2^/df) = 2.816, goodness of fit index (GFI) = 0.977, adjusted-goodness-of-fit index (AGFI) = 0.918, root mean square error of approximation (RMSEA) = 0.078, comparative fit index (CFI) = 0.986, incremental fit index (IFI) = 0.986, Tucker Lewis index (TLI) = 0.960, and Normed-fit index (NFI) = 0.978. These values indicated that the model fitted well.

#### Mediating effect test

The Bootstrap analysis method was used for model verification, with the number of iterations set at 5000 times and the confidence interval of 95%. The results showed that the 95% confidence intervals of the direct and indirect effects of workplace bullying on compassion satisfaction, burnout, and secondary trauma stress did not contain zero [34]. This indicates that coping styles played a partial mediating effect between workplace bullying and the pairwise relationships of compassion satisfaction, burnout, and secondary trauma stress. The mediating effects and the ratio of relative mediation effect of positive coping style and negative coping style between workplace bullying and the three dimensions of nurses' ProQOL are shown in Table 3.

**Table 3:** Effect of mediating model (standardization)

Dependent Variable	Effect	Model pathways	Standardized effect (*β*)	*SE*	*95% CI* LL	*95% CI* UL	Relative mediating effect (%)
CS	DE	WPB → CS	-0.160	0.068	-0.299	-0.008	37.21
	IE	WPB → PCS → CS	-0.161	0.016	-0.181	-0.119	37.44
		WPB → NCS → CS	-0.109	0.029	-0.160	-0.045	25.35
	TE	—	-0.430	0.055	-0.530	-0.316	
BO	DE	WPB → BO	0.164	0.057	0.026	0.298	33.81
	IE	WPB → PCS → BO	0.164	0.039	0.090	0.244	33.81
		WPB → NCS → BO	0.157	0.046	0.075	0.254	32.38
	TE	—	0.485	0.046	0.388	0.570	
STS	DE	WPB → STS	0.167	0.065	0.034	0.298	37.28
	IE	WPB → PCS → STS	0.105	0.037	0.038	0.184	23.43
		WPB → NCS → STS	0.176	0.043	0.017	0.186	39.29
	TE	—	0.448	0.044	0.358	0.530	

① *DE* Direct effect

② *IE* Indirect effect

③ *TIE* Total indirect effect

④ *TE* Total effect

## Discussion

### Analysis of the current situation of workplace bullying, coping styles, and professional quality of life among nurses

In this study, the nurses' workplace bullying score was 38.72 ± 12.30, at a moderate level, which is higher than the previous research results in Taipei by Shen et al. [20], and slightly higher than the previous research results in China [35]. According to the findings of our investigation, out of 297 clinical nurses, 176 of them were identified as victims of bullying, accounting for 59.3% of the total, which indicates a high proportion in comparison to the global range mentioned earlier. Among them, 72 individuals experienced severe bullying, accounting for 24.2%. The reason for this is that participants are exclusively from tertiary Grade A hospitals, which represent the highest level of hospitals in China's hierarchical classification system. These hospitals provide high-quality medical and healthcare services and undertake tasks such as education and research. Tertiary Grade A hospitals have advanced medical expertise with comparable expenses to general hospitals, accommodating a larger patient population and bearing heavier workloads. Nurses in these hospitals commonly face high workloads, job demands, and long-term high-pressure environments, leading to heightened mental stress. Insufficient communication contributes to misunderstandings and bullying occurrences [36, 37]. Furthermore, our study observed that 52.9% of the nurses were below the age of 25, while 68.7% had less than five years of work experience. Notably, 18.5% of the respondents held a master's degree or higher, which is considerably higher than the proportions of 1.7% [38] and 3.9% [16] reported in previous studies. This difference in age and education level could potentially contribute to the relatively high prevalence of bullying identified in our study. Previous research has demonstrated that workplace bullying rates vary across age groups and educational backgrounds. Young and inexperienced nurses, as well as those with advanced degrees, tend to be more susceptible to becoming targets of workplace bullying compared to their more experienced counterparts [39]. One possible explanation for this pattern is the increasing prevalence of higher education among the younger generation of nurses, resulting in a generally higher level of confidence and openness [40]. However, this can be perceived as arrogance by some older nurses who may worry about their professional status and fear being surpassed in terms of salary and position [41]. These concerns can lead to jealousy and resentment, contributing to workplace bullying. Additionally, traditional Chinese concepts of obeying authority, respecting seniority, and emphasizing hierarchy further compound the workplace oppression experienced by young nurses [42]. They are often burdened with additional tasks and responsibilities, making them particularly vulnerable to becoming primary victims of workplace bullying.

In this study, the nurses' positive coping style score was 34.59 ± 5.72, which was at a medium to high level and higher than the results before the study [43, 44]. The score for negative coping style was 20.34 ± 5.08, which was at a middle level and lower than the study of Yan Chen [45], but higher than the results of Cheng [46]. The current situation of heavy nursing workload and pressure has attracted the attention of hospitals and the nursing industry. Training and seminars on improving nurses' coping ability and cognitive level have been gradually carried out, which is beneficial to improving nurses' ability to cope with conflict and emotional management [47]. Since the outbreak of the COVID-19 epidemic, people's understanding and support for healthcare personnel have continued to deepen, and the sense of the professional value of nurses has also been enhanced, which is conducive to nurses maintaining a positive response at work.

In this study, the scores for the three dimensions of a nurse's ProQOL were as follows: compassion satisfaction 27.56 ± 4.79, burnout 30.51 ± 4.33, and secondary traumatic stress 28.42 ± 4.65, all of which were at a medium level. The compassion satisfaction score was consistent with a study in China [48], but lower than the research results of Jialin Wang [49] and Emel Gümüş [50]. The scores of burnout and secondary trauma stress were slightly higher than the results of previous studies [7, 51]. Nursing is a rewarding profession that involves assisting others. Nurses experience happiness and satisfaction in the process of providing care services for patients, which helps to maintain a high level of compassion satisfaction [49]. However, in recent years, the implementation of the concept of high-quality nursing service and increasing demands from patients and their families have made nurses' work more extensive and intensive. This has led to an increase in the conflict between the supply and demand of nursing services, as well as an increase in the contradiction between doctors and patients. These factors are likely to lead to burnout among nurses [52]. In addition, due to the unique working environment and service objects, nurses often encounter pain, depression, rescue, or death of patients at work. This can lead to nurses easily experiencing the impact of negative emotions at work, resulting in secondary traumatic damage [1, 53]. Therefore, nursing managers should closely monitor nurses' psychology and working conditions, alleviate their work pressure, and enhance their ProQOL.

### Correlation between workplace bullying, coping styles, and ProQOL among nurses

The results of this study showed that workplace bullying among nurses was negatively correlated with compassionate satisfaction and positively correlated with burnout and secondary trauma stress, thereby confirming hypotheses 1 and 2. These findings indicate that the more severe the workplace bullying that nurses experienced, the less pleasure and satisfaction they felt at work, and the higher the level of burnout and secondary trauma stress they incurred. These findings are consistent with the study by Jie Peng [16], which indicated that long-term exposure to bullying work environments, such as harassment, offense, and isolation, would increase nurses' physical and mental consumption, leading to a decrease in their work accomplishment and compassion satisfaction. At the same time, it would exacerbate mental and emotional burnout, helplessness, anxiety, and other adverse psychological reactions, and aggravate the secondary trauma of nurses [16, 24]. Workplace bullying was negatively correlated with positive coping style and positively correlated with negative coping style. This means that the more serious workplace bullying the nurses suffer, the more likely they are to adopt negative coping style instead of positive ones. It indicates that bullying in the workplace not only damages the physical and mental health of nurses but also adversely affects their normal nursing work and interpersonal communication [41]. This can cause them to lose their enthusiasm for work and life, and can easily lead to insufficient working motivation and mistakes [38]. Positive coping style was found to have a positive correlation with compassion satisfaction and a negative correlation with burnout and secondary trauma stress. Conversely, negative coping styles showed a negative correlation with compassion satisfaction and a positive correlation with burnout and secondary trauma stress. These results imply that higher levels of positive coping are associated with increased compassion satisfaction among nurses, while higher levels of negative coping are linked to more severe burnout and secondary trauma stress. These findings align with previous research [45], highlighting the importance for nurses to adopt positive coping strategies at work to enhance their ProQOL and overall well-being.

### The mediating role of coping styles between workplace bullying and professional quality of life among nurses

The results of this study showed that coping styles played a partial imediating role between workplace bullying and compassion satisfaction, burnout, and secondary trauma stress of nurses, respectively. Workplace bullying, as a persistent form of aggression and insults, is a stressor for the victims [9]. Previous research has shown that victims tend to adopt a positive coping style in the early stages of bullying by finding the silver lining, maintaining a good mood and behavior, and seeing the challenges as opportunities for personal growth [54]. This positive coping style can help nurses maintain a high level of compassion satisfaction in the early stages of workplace bullying [55]. However, long-term exposure to a bullying environment may lead to social alienation and coping fatigue among nurses. They are more likely to use negative avoidance instead of positive coping styles when faced with difficulties [26]. This negative coping style can lead to self-doubt, deterioration of mental health, lower quality of life, and increased empathy fatigue at work [56]. Our findings align with previous research, suggesting that nurses may attempt to minimize their pain by avoiding and refusing due to persistent bullying, which can result in burnout and high levels of secondary trauma stress. Zhu's [57] study on medical students in China highlighted the significant role of coping style in protecting individuals' physical and mental health as an intermediary mechanism between stress and well-being.

## Practical implications

Bullying in the nursing field has caused significant harm to nursing practitioners in various countries and has seriously affected the ProQOL of nurses. This study verifies that choosing positive or negative coping style to face workplace bullying would have completely different effects on the ProQOL of nurses. Nursing managers should have a comprehensive understanding of the harm that workplace bullying poses to both the organization and individuals. They should actively create a supportive work environment for nurses, establish a robust system for reporting and addressing bullying incidents, and minimize workplace bullying. In addition, nursing personnel should receive education on recognizing and coping with workplace bullying, enabling them to better identify and respond to such behaviors, thereby reducing the harm caused by bullying and ultimately enhancing their ProQOL.

## Limitation

Firstly, one of the limitations of this study was based on the participants' self-reports. All participants have gained anonymity and confidentiality, but they still can't completely avoid the reaction bias. In addition, the sample size of this study was small, including only nurses from two tertiary grade A hospitals in Wuhan, and 52.9% of the participants are under the age of 25, which limited the universality of the research results. In future research, a multi-center investigation should be carried out to further verify the conclusion of this study. Finally, because this study was a cross-sectional study design, we couldn’t find the causal relationship between the study variables. Therefore, more longitudinal research designs are needed in the future to explore the influence of workplace bullying and coping styles on nurses' professional quality of life.

## Conclusion

To sum up, workplace bullying and the three dimensions of nurses’ ProQOL in this study are at a medium level. Workplace bullying could directly have a negative impact on nurses' compassionate satisfaction, and aggravate nurses' burnout and secondary trauma stress. Coping styles play an mediating role in workplace bullying and nurses' ProQOL. Positive coping style can increase nurses' compassion satisfaction and reduce the influence of bullying on burnout and secondary trauma stress. Negative coping style will aggravate burnout and secondary trauma stress, and reduce nurses' level of compassion satisfaction. Managers should adopt corresponding strategies to reduce workplace bullying, help improve nurses' positive coping style level, and then improve their ProQOL.

## Data Availability

The data that support the findings of this study are available from the corresponding author on reasonable request.

## References

[1] Liu S, Chen SJ, Zou XM, Sun ZW, Huang ZY (2021). Research progress of clinical nurses’ current status of professional quality of life and influencing factors. Chin J Nurs Train.

[2] Stamm BH: The Concise ProQOL Manual: The concise manual for the Professional Quality of Life Scale, 2nd Edition.; 2010. Retrieved from https://nbpsa.org/images/PRP/ProQOL_Concise_2ndEd_12-2010.pdf

[3] Jiang W, Zhao X, Jiang J, Zhou Q, Yang J, Chen Y, Goldsamt L, Williams AB, Li X (2021). Hospital ethical climate associated with the professional quality of life among nurses during the early stage of COVID-19 pandemic in Wuhan, China: a cross-sectional study. Int J Nurs Sci.

[4] Pu HY. Investigation and correlation analysis of psychological capital, professional quality of life and subjective well-being of standardized training nurses. LuZhou: (Master), China Southwest medical university; 2019.

[5] Cruz JP, Alquwez N, Mesde JH, Almoghairi AMA, Altukhays AI, Colet PC (2020). Spiritual climate in hospitals influences nurses’ professional quality of life. J Nurs Manage.

[6] Wang AX, Wang BQ (2020). Status quo and the influencing factors of empathy fatigue of clinical nurses. Chin Nurs Res.

[7] OzgeSukut GSCH (2022). Professional quality of life and psychological resilience among psychiatric nurses. Perspect Psychiatr C.

[8] Boni RL, Dingley C, Reyes A (2022). Measuring professional quality of life in nurses: a realist review. Clin J Oncol Nurs.

[9] Obeidat RF, Qan Ir Y, Turaani H (2018). The relationship between perceived competence and perceived workplace bullying among registered nurses: a cross-sectional survey. Int J Nurs Stud.

[10] Parchment J, Andrews D (2019). The incidence of workplace bullying and related environmental factors among nurse managers. J Nurs Adm.

[11] Van den Brande W, Baillien E, Elst TV, De Witte H, Godderis L (2019). Coping styles and coping resources in the work stressors–workplace bullying relationship: a two-wave study. Work Stress.

[12] Al Omar M, Salam M, Al-Surimi K (2019). Workplace bullying and its impact on the quality of healthcare and patient safety. Hum Resour Health.

[13] Bae S, Hong H, Chang J, Shin S (2021). The association between Korean clinical nurses’ workplace bullying, positive psychological capital, and social support on burnout. Int J Env Res Pub He.

[14] Serafin LI, Czarkowska-Pączek B (2019). Prevalence of bullying in the nursing workplace and determinant factors: a nationwide cross-sectional Polish study survey. BMJ Open.

[15] Ng CS, Chan VC (2021). Prevalence of workplace bullying and risk groups in Chinese employees in Hong Kong. Int J Env Res Pub He.

[16] Peng J, Luo H, Ma Q, Zhong Y, Yang X, Huang Y, Sun X, Wang X, He J, Song Y (2022). Association between workplace bullying and nurses' professional quality of life: the mediating role of resilience. J Nurs Manage.

[17] Brewer G. Workplace bullying in healthcare professions. Int J Occup Health Public Health Nurs. 2015;2(01):11–28.

[18] Guo J, Zhang BH, Huang LX, Zheng XY, Wu QH (2015). Bullying in workplace: an explorative study. Chin J Clin Psychol.

[19] Rahman CS, Humayun K, Nahida A, Azmain IM, Kumar RA, Rahman CM, Ahmed H. Impact of workplace bullying and burnout on job satisfaction among Bangladeshi nurses: a cross-sectional study. Heliyon. 2023;9(2):e13162. 10.1016/j.heliyon.2023.e13162 PMC9900271 36755612

[20] H STS, Ching MS, Liu GS, Chiu KC, Chen TJ, Huey CM, Huang HC. The role of workplace bullying in the relationship between occupational burnout and turnover intentions of clinical nurses. Appl Nurs Res. 2022;68:151483. 10.1016/j.apnr.2021.151483 34629280

[21] Meng YX, Li Y, Zhou CL, Li J (2021). The mediating role of positive coping style in the relationship between social support and psychological resilience of medical staff. Chin Med Equip.

[22] PorteroDeLa Cruz S, Cebrino J, Herruzo J, Vaquero-Abellán M (2020). A multicenter study into burnout, perceived stress, job satisfaction, coping strategies, and general health among emergency department nursing staff. J Clin Med.

[23] Zhou H, Peng J, Wang D, Kou L, Chen F, Ye M, Deng Y, Yan J, Liao S (2017). Mediating effect of coping styles on the association between psychological capital and psychological distress among Chinese nurses: a cross-sectional study. J Psychiatr Ment Health Nurs.

[24] Wang Y, Wang P (2019). Perceived stress and psychological distress among Chinese physicians: the mediating role of coping style. Medicine (Baltimore).

[25] Zhang Y. A Study on the influences of stress coping style on job burnout in college teachers: the moderating effect of organizational support. Yan Ji: (Master), China Yanbian University; 2018.

[26] Zhou LL. The relationships among professional quality of life, work engagement and coping style of nurses and its intervention. ChongQing, China: (Master), China Southwestern University; 2020.

[27] Zheng WJ, He F (2020). Sample size estimate for cross-sectional study. Prev Med.

[28] Kline RB (2015). Principles and practice of structural equation modeling.

[29] Einarsen S, Hoel H, Notelaers G (2009). Measuring exposure to bullying and harassment at work: validity, factor structure and psychometric properties of the negative acts questionnaire-revised. Work Stress.

[30] Xun HJ, Liu HX, Tian ZL (2012). A preliminary reliability and validity study of Chinese version of the negative acts questionnaire revised. Chin Nurs Manage.

[31] Zheng X, Yang M, Gao W, Chen FF (2013). The Chinese professional quality of life scale: testing of reliability and validity in nurses. Chin J Nurs Sci.

[32] Xie YN (1998). Reliability and validity of the simplified coping style questionnaire. Chin J Clin Psychol.

[33] Tang DD, Wen ZL (2020). Statistical approaches for testing common method bias: problems and suggestions. Psychol Sci.

[34] Wen ZL, Ye BJ (2014). Analyses of mediating effects: the development of methods and models. Adv Psychol Sci.

[35] Sun YQ, Ge YX, Ke ZW, Li YY, Jin QX, Lu YF (2018). Effect of workplace bullying on post-traumatic stress disorder in nursing staff. Chin J Industr Hygiene Occup Dis.

[36] Niu A, Li P, Duan P, Ding L, Xu S, Yang Y, Guan X, Shen M, Jiang Y, Luo Y (2022). Professional quality of life in nurses on the frontline against COVID-19. J Nurs Manag.

[37] Kim K, Han Y, Kim J (2015). Korean nurses’ ethical dilemmas, professional values and professional quality of life. Nurs Ethics.

[38] Chen J, Zheng YN (2020). Workplace bullying and its influencing factors among clinical nurses. Chin J Nurs Sci.

[39] Kim Y, Choi JS (2021). Individual and organizational factors influencing workplace cyberbullying of nurses: a cross-sectional study. Nurs Health Sci.

[40] Clendon J, Walker L (2012). 'Being young': a qualitative study of younger nurses' experiences in the workplace. Int Nurs Rev.

[41] Shorey S, Wong PZ (2021). A qualitative systematic review on nurses'' experiences of workplace bullying and implications for nursing practice. J Adv Nurs.

[42] Chen J, Zheng YN (2020). Research progress on workplace bullying in the field of nursing. Chin Nurs Manage.

[43] Li W, Yuan P, Sun J, Xu M, Wang Q, Ge D, Jiang M, Xing L, Du W, Li Q (2022). Resilience, coping style, and COVID-19 stress: effects on the quality of life in frontline health care workers. Psychol Health Med.

[44] Wang Y, Liu CL (2019). The mediating effect of social support and coping style between nurses' personality characteristics and mental health. Chin J Nurs Educ.

[45] Chen Y, Song B, Fu X (2021). The improvement of organizational psychological support of medical staff corresponds to the impact on emergencies. Chin J Qilu Nurs.

[46] Cheng L, Yang J, Li M, Wang W (2020). Mediating effect of coping style between empathy and burnout among Chinese nurses working in medical and surgical wards. Nurs Open.

[47] Zhang W, Sun FF, Lu QH (2019). Role of self-efficacy in the relationship between self-acceptance and positive coping style of psychiatric nurses. Chin J Pract Nurs.

[48] Xiao DJ, Xiao HY, Wu YS, Li HY, Chen HY (2020). Influence of work addiction on nurses’ professional quality of life. Chin Modern Clin Nurs.

[49] Wang J, Okoli C, He H, Feng F, Li J, Zhuang L, Lin M (2020). Factors associated with compassion satisfaction, burnout, and secondary traumatic stress among Chinese nurses in tertiary hospitals: a cross-sectional study. Int J Nurs Stud.

[50] Gumus E, Alan H, Taskiran Eskici G, EskinBacaksiz F (2021). Relationship between professional quality of life and work alienation among healthcare professionals. Florence Nightingale J Nurs.

[51] Liu MH, Qin HY (2020). Current status of professional quality of life among oncology nurses and its influence factors: a 210-case study. J Nurs (China).

[52] Chen L, Cao XY, Chen YH, Zhang L, Shi L (2017). Relationship between empathy fatigue and job involvement among hemodialysis nurses. Chin J Nurs Sci.

[53] Maila S, Martin PD, Chipps J (2020). Professional quality of life amongst nurses in psychiatric observation units. S Afr J Psychiatr.

[54] Tsuno K (2022). Do personal resilience, coping styles, and social support prevent future psychological distress when experiencing workplace bullying? Evidence from a 1-year prospective study. BMC Psychol.

[55] Yoo SY, Ahn HY (2020). Nurses' workplace bullying experiences, responses, and ways of coping. Int J Env Res Pub He.

[56] Rotman M, Andela CD, Majoor BC, Dijkstra PD, Hamdy NA, Kaptein AA, Appelman-Dijkstra NM. Passive coping strategies are associated with more impairment in quality of life in patients with fibrous dysplasia. Calc Tissue Int 2018(No.5):469–475. 10.1007/s00223-018-0441-1 PMC6182587 29948062

[57] Zhu X (2021). Mediating role of coping style between social support and basic psychological needs among medical and nursing students. Chin Nurs Res.

